# Development of a questionnaire weighted scoring system to target diagnostic examinations for asthma in adults: a modelling study

**DOI:** 10.1186/1471-2296-5-30

**Published:** 2004-12-17

**Authors:** Sybil Hirsch, Timothy L Frank, Jonathan L Shapiro, Michelle L Hazell, Peter I Frank

**Affiliations:** 1 General Practice Research Unit, North West Lung Research Centre, Wythenshawe Hospital, Manchester, M23 9LT, UK; 2 Department of Computer Science, University of Manchester, Oxford Road, Manchester M13 9PL, UK

## Abstract

**Background:**

Identification and treatment of unrecognised asthmatics in the community is important for improving the health of the individual and minimising cost and quality of life burden. It is not practical to offer clinical diagnostic assessment to whole communities, and a simple tool such as a questionnaire is required to identify a smaller target group. Conventional questionnaire screening methods which separate individuals into positive and negative categories have resulted in large numbers of individuals requiring clinical assessment. This study has therefore developed and tested a weighted scoring system that prioritises those most urgently in need, based on their questionnaire responses.

**Methods:**

A stratified random sample of adult respondents to a general practice postal questionnaire survey were categorised 'asthmatic' or 'non-asthmatic' according to three expert physicians' opinions. Based on this categorisation, logistic regression was used to derive weights reflecting the relative importance of each question in predicting asthma, allowing calculation of weighted scores reflecting likelihood of asthma. Respondents scoring higher than a chosen threshold would be offered diagnostic examination.

**Results:**

Age and presence of wheeze were most influential (weight 3) and overall weighted scores ranged from -1 to 13. Positive predictive values (PPV) were estimated. For example, setting the threshold score at nine gave an estimated PPV for asthma diagnosis of 93.5%, a threshold score of seven corresponded to PPV 78.8%. PPV estimates were supported by examining 145 individuals from a new survey.

**Conclusion:**

Weighted scoring of questionnaire responses provides a method for evaluating the priority level of an individual 'at a glance', minimising the resource wastage of examining false positives.

## Background

There are individuals in the community who are asthmatic but are not receiving treatment because they are unknown to the medical services [1-5]. Detecting these unrecognised asthmatics may be important for short-term health, prevention of long term airway remodelling and minimisation of cost and quality of life burden [6]. The reliable diagnosis of asthma, however, requires full clinical assessment [7]. Since it is clearly not practical in terms of resource allocation to offer this to whole communities, a simple tool such as a questionnaire is needed to identify a smaller target group. Conventional targeting approaches which separate individuals into 'positive' and 'negative' categories have failed to reduce the number of potential examinations to a manageable level. For example, in the Wythenshawe Community Asthma Project (WYCAP) [8-10], respondents who had four or more 'yes' answers to six key questions on a postal respiratory questionnaire were deemed possible asthmatics in need of clinical assessment. However, this method identified approximately 350 individuals for examination per practice. A more refined screening method, which could identify more than two categories, would allow the screening threshold to be adjusted to take into account available resources.

Defining additional categories according to the possible numbers (0 to 6) of 'yes' answers to the 6 key questions is too simplistic as it assumes equal weights for each question and takes no account of variables such as age and gender. The present paper describes the development of a more refined method, which includes additional questions and uses question weights to calculate a score for each respondent. This weighted score reflects probability of asthma, allowing clinical resources to be targeted to those most likely to have the disease. A score is chosen as a threshold and the questionnaire is given in an opportunistic fashion to patients consulting a medical practice for any reason. The questionnaire score is calculated 'at a glance' and those whose score is higher than the chosen threshold, and who are not already receiving asthma treatment, are offered diagnostic examination. The threshold can be adjusted to take resource limitations into account.

## Methods

The study described in this paper was based on data collected as part of the WYCAP investigation. WYCAP is a long term prospective investigation of the natural history of asthma in two general practice populations. It is based on a postal respiratory questionnaire adapted from the European Community Respiratory Health Questionnaire (ECRHQ) [11]. Four postal questionnaire surveys of all adults (16 years and over) registered to the two practices were conducted in 1993, 1995, 1999 and 2001. Additional data were obtained in 1995 and 2001 by invitation of selected respondents for clinical review. Ethical approval for the present study was obtained from South Manchester Local Research Ethics Committee and statistical analysis was performed using SPSS for Windows [12]. The study uses questionnaire responses and additional data obtained as part of the 1995 and 2001 adult surveys and the investigations were conducted in three phases. First, a weighted scoring system was developed based on a subset of adult questionnaires and clinical review data collected in 1995. Next, this scoring system was applied to the questionnaires from the whole population of respondents to the 1995 survey in order to estimate its positive predictive value (PPV) in finding undiagnosed asthmatics in the community. Finally, the validity of the scoring system was tested in a new data set in the form of a subset of questionnaires and clinical review data selected from the 2001 WYCAP survey.

### Phase 1: Development of the weighted scoring system

It was necessary to select an asthma-enriched sample in order to determine response patterns of both asthmatic and non-asthmatic respondents [13]. Respondents from the 1995 survey were stratified based on the number of 'yes' answers (0, 1–3, 4, 5 and 6) to six key questions on the postal questionnaire. These were questions which were believed to be important in the detection of an asthmatic, namely questions relating to symptoms in the last 12 months (wheezing, chest tightness, shortness of breath, night cough), family history of asthma and associated conditions of hayfever or eczema [see Additional file 1]. The target numbers selected from each stratum were based on clinical intuition and aimed to provide a stratified random sample with approximately equal numbers of asthmatics and non-asthmatics. Individuals were selected from one of the practices and invited for clinical review by the research clinician, including full history, physical examination, spirometry, a test for reversibility to beta-2 agonists, bronchial challenge with histamine, electronic peak flow diaries and skin prick testing to five common allergens. The clinical review results were sent separately to three expert respiratory physicians who had no knowledge of the questionnaire responses. Each physician was asked to rate the probability of asthma as <50%, 50–90%, or >90%. Bayesian methods were used to combine the physicians' opinions into a consensus estimate of probability of asthma for each individual [14]. In order to ensure the independence of the expert opinions required by these methods, the physicians did not confer with each other and were not given any diagnostic criteria or guidelines. Those reviewed individuals in whom the consensus estimate of probability of asthma was 50% or more were designated clinically asthmatic and the remainder were designated clinically non-asthmatic. By modelling the relationship between questionnaire responses and clinical asthma status, a weight was determined for each question, reflecting its importance in predicting asthma status of the respondent. For each questionnaire, the weights for the relevant responses ('yes' answers) were summed to produce an asthma score reflecting the probability of asthma in that individual.

The weights were derived based on a linear logistic regression model, designed to take as input the questionnaire responses 1 to 11 and predict the probability of asthma for any individual from the wider community. The model associates each question on the questionnaire with a coefficient reflecting the relative importance of that question in determining probability of asthma. A positive value for a coefficient influences towards a decision that the individual is classified asthmatic, the higher the value the greater the effect. Negative coefficients influence away from a decision that the individual is classified asthmatic. In order to provide a model representative of the wider community, a cross-validation technique was used [15] in which several candidate logistic regression models were produced and their respective coefficients averaged. These average values were rounded to the nearest whole number to give the question weights prior to summing to produce the weighted scores.

### Phase 2: estimating positive predictive value

The full set of postal responses to the 1995 questionnaire survey was used to assess effectiveness by estimating PPV of the scoring system in detecting undiagnosed asthmatics in the community. Weighted scores were calculated for all 1995 survey respondents and evidence of either 'asthma diagnosis ever' or 'prescription for asthma medication in the last 12 months' was obtained from their medical records. The percentage of undiagnosed asthmatics with a threshold score or higher, who could be expected to be confirmed clinically asthmatic, was estimated using a technique which referenced the probabilities of asthma of sample respondents, (based on the combined expert opinions), their population strata (based on number of 'yes' answers to the six key questions) and weighted scores (based on questionnaire responses) [see Additional file 2]. This estimation was repeated for various score thresholds. These expected percentages provided estimates of the positive predictive values of the score thresholds [16].

### Phase 3: testing the validity of the weighted scoring system in a new data set

The estimates of PPV for the various score thresholds depend on the fact that higher scores correspond to higher probability of asthma. The weighted scoring system is valid if it can be shown to retain this property when applied to a new data set, and this was tested by comparing scores with clinical diagnosis of asthma in a subset of data collected in 2001. Sophisticated modelling techniques had been used to rank the 2001 survey questionnaires in order of probability of asthma as part of another study [17,18]. The test subset for this study consisted of questionnaires from the top 10% of this ranked list (most likely asthmatics) and a random sample (approximately 1 in 5) of low probability individuals selected at random from the end 200 of the population list. As in 1995, each selected individual was invited for clinical review and the results were sent separately to another three respiratory physicians. The purpose of the low probability individuals was simply to illustrate the diagnostic discrimination of the physicians.

The method of combining the three independent physician opinions differed from that used in phase 1. This time, the aim was not to produce diagnoses as the basis of a mathematical model, but rather to emulate as far as possible diagnosis in a clinical setting. Each physician was therefore asked, on the basis of the clinical review information, to classify each individual as 50% or greater probability of obstructive airways disease (OAD), or less than 50% probability. For those 50% or greater probability (more likely than not to have OAD), the physician was asked to state whether the disease was asthma, chronic obstructive pulmonary disease (COPD) or mixed disease (asthma and COPD). Individuals in whom at least two physicians diagnosed asthma or mixed disease were designated clinically asthmatic.

As part of the 2001 population survey, each reviewed individual had already completed a postal questionnaire, and a weighted score was calculated based on questionnaire responses. For each possible score, the proportion of reviewed individuals with that score or higher, who had been designated clinically asthmatic was calculated. These percentages reflect the association between score and probability of asthma and were used as an indication of the validity of the postal questionnaire and scoring system.

For comparison, unweighted scores (number of 'yes' answers to the six key questions) were also calculated for respondents from the top 10% of the ranked population list, and the percentage of asthmatics associated with each of the unweighted score thresholds was similarly calculated.

## Results

In the 1995 WYCAP survey, 10429 individuals were sent a postal respiratory questionnaire with a 72.7% response rate, leaving 6825 after exclusion of incomplete questionnaires. Four hundred and twenty individuals were selected for the stratified random sample and invited for clinical review. Of these, 201 (48%) attended, leaving 180 after exclusion of those whose screening questionnaire was incomplete. The ages of the 180 respondents with fully completed questionnaires ranged from 16 to 83 years, with a median age of 50.5 years, and 41.7% were male. Eighty four individuals were designated clinically asthmatic and 96 non-asthmatic based on the combined opinions of the three physicians.

### Phase 1

The weights derived for each question are shown in table 1. The features most influential towards classifying an individual as asthmatic (weight 3) were reporting of wheeze in the last 12 months and being in age group 1 (16–34 years). Reporting an asthma attack in the last 12 months and being in age group 2 (35 – 54 years) were next most influential with weights of 2. Cigarette smoking (weight -1) was a small influence away from a classification as asthmatic. Under this scoring system, the responses to questions 5 and 6 (woken by an attack of shortness of breath or coughing) were found to be zero weighted and therefore, when used in combination with this set of questions, did not contribute further to the overall weighted score. The questions relating to breathlessness whilst wheezing and wheeze in the absence of a cold (questions 3.1 and 3.2) are conditional on the reporting of wheeze at any time (question 3) and therefore although the model for the prediction of asthma probability requires them, it is difficult to place a clinical interpretation on their values.

**Table 1:** Weights associated with each question on the questionnaire

Question Number	Variable based on question	Scoring system weights
1	*Age (16 – 34 years)	3
1	*Age (35 – 54 years)	2
1	*Age (55 – 74 years)	1
2	Sex male	1
3	**Wheeze in last 12 months	3
3.1	Breathless whilst wheezing	0
3.2	Wheeze in absence of cold	-1
4	**Woken by chest tightness in last 12 months	1
5	**Woken by shortness of breath in last 12 months	0
6	**Woken by night cough in last 12 months	0
7	Asthma attack last 12 months	2
8	Currently taking asthma medication	1
9	**Family history of asthma	1
10	**Hayfever/eczema ever	1
11	Smoker	-1

* Age was aggregated into 4 categories. The 4th age category (>= 75) years is excluded from the model because it is dependent on the other 3

** Key variables used in simple scoring of 'yes' answers to key questions

### Phase 2

Weighted scores were calculated for all postal questionnaires (n = 6825) in the 1995 WYCAP population and ranged from -1 to 13. Table 2 shows the percentage of previously undiagnosed asthmatics expected to be found when using the various scores as thresholds, that is, recommending a clinical review for all individuals with that score or higher. A score of nine or more was found in 335 individuals. Of these, 80 had no evidence of asthma diagnosis in their medical records and it would be expected that 75 (93.5%) would be diagnosed clinically asthmatic. Lowering the score threshold below nine progressively decreased the expected efficiency, so that examining those scoring eight or more increases the number of false positives with only 82.5% expected to be confirmed asthmatic. The 4537 individuals scoring four or less are much less likely to be asthmatic (expected percentage of asthmatics in this group was 5%).

**Table 2:** Positive predictive value estimates for weighted scoring applied to all respondents to 1995 postal survey (n = 6825)

Threshold Score	Number of individuals above threshold	Number eligible for review (no asthma diagnosis)	Undiagnosed individuals expected to be diagnosed clinically asthmatic	
			Number	*Percent *(Positive predictive value)
11	76	13	12	*90.5*
10	178	33	31	*94.2*
9	335	80	75	*93.5*
8	577	195	161	*82.5*
7	891	394	310	*78.8*
6	1289	715	437	*61.2*
5	2098	1427	678	*47.5*
4	3332	2592	769	*29.7*
3	4667	3874	894	*23.1*
2	5769	4938	929	*18.8*
1	6524	5670	933	*16.5*
All respondents	6825	5964	934	*15.7*

### Phase 3

The third phase confirmed that higher scores correspond to higher probabilities of asthma in the independent test subset selected from the 2001 survey respondents. Two hundred and eighty three individuals comprised the top 10% of the probability ranked list and were invited for review along with 39 low probability individuals selected from the end 200. In total, 145 individuals attended for review, their ages ranged from 16 to 91 years with a median age of 50 years, and 40.7% were male.

One hundred and twenty six high probability and 19 low probability individuals attended. For the 126 high probability asthmatics from the top 10% of the ranked list, scores ranged from six to 12, compared with a maximum score of eight in those respondents not in the top 10%. The age range and median age associated with each score threshold is reported in table 3, which shows that lower score thresholds are associated with increasing age.

**Table 3:** Percentage of individuals with threshold scores or higher who were found clinically asthmatic in the 2001 validation set (n = 126)

Threshold Score	Number of individuals above threshold	Median age (years) *[range]*	*Majority verdict asthma*	*Majority verdict mixed disease*	Majority verdict asthma or mixed disease	
					Number	*Percent*
11	11	33 *[21–54]*	*9*	*1*	10	90.9
10	32	40.5 *[21–74]*	*27*	*1*	28	87.5
9	72	44.5 *[18–75]*	*53*	*10*	63	87.5
8	114	47 *[17–88]*	*76*	*19*	95	83.3
7	121	47 *[17–88]*	*77*	*19*	96	79.3
6	126	47 *[17–88]*	*81*	*20*	101	80.1

In general, agreement between the experts was good with pair-wise kappa statistics of 0.62, 0.64 and 0.65 respectively. All three experts agreed the same diagnostic category for 99 out of the 145 reviewed individuals (82 from the high probability group and 17 from the low probability group) and two out of three experts agreed for a further 38 (37 high probability and one low probability). The percentages of individuals diagnosed as having asthma or mixed disease associated with each score threshold are shown in table 3. The higher the questionnaire score the higher the probability of asthma in the respondent. Of the 72 individuals scoring nine or more, 63 (88%) were clinically diagnosed as having asthma or mixed disease (53 asthma, 10 mixed). Lowering the threshold reduces the percentage of asthmatics diagnosed, such that of the 121 individuals scoring seven or more only 96 (79%) were clinically diagnosed as having asthma or mixed disease (77 asthma, 19 mixed). The 19 low probability individuals selected from the end 200 of the ranked list all achieved scores of zero or one and 18 were diagnosed clinically non-asthmatic based on the physician opinions.

Table 4 shows the percentage of clinical asthmatics associated with each of the non-weighted score thresholds, illustrating that ranking according to non-weighted scores does not demonstrate the ranking in order of probability of asthma that is achieved by the weighted scoring system.

**Table 4:** Percentage of individuals with non-weighted threshold scores or higher who were found clinically asthmatic in the 2001 validation set (n = 126)

**Number 'yes' answers out of 6 key questions (screening threshold)**	**No. individuals above threshold**	**Number (*percentage*) diagnosed asthma or mixed disease**
6	30	25 (*83.3%*)
5	74	64 (*86.5%*)
4	103	86 (*83.5%*)
3	119	98 (*82.4%*)
2	124	100 (*80.6%*)
1	126	101 (*80.1%*)

## Discussion

In general, questionnaire surveys for detecting individuals likely to have a disease partition respondents into 'positive' and 'negative' categories. This is appropriate when a community-wide survey can be undertaken and the medical services are able to provide clinical assessment for all those in the 'positive' category. This study has developed a method for optimising limited resources by targeting expensive clinical examinations to those most likely to be at risk, minimising the chance of needlessly examining a healthy individual and allowing resource availability to be taken into account.

Developing a model which uses a questionnaire to identify likely asthmatics in the general population crucially depends on having a subset of questionnaires from respondents reliably classified as 'asthmatic' or 'non-asthmatic' on which to build the model. In a condition such as asthma there is inherent uncertainty in the diagnosis, and even detailed clinical review information inevitably produces disagreement between experts as to the diagnostic category for some of the reviewed individuals. Other studies have produced questionnaire models for predicting high risk asthmatics [4,9,10,19] and these studies have either used a single expert or resolved the problem of disagreement between experts using methods such as 'majority verdict' or designated diagnostic rules. However, where more than one expert is involved, even permitting the experts to discuss problem cases did not always result in complete agreement [4]. The importance of this present study lies in the rigorous methods used to capture the uncertainty inherent in asthma diagnosis by combining the opinions of three experts using standard statistical techniques. These aimed to produce a reliable diagnosis for each individual in the phase 1 subset of questionnaires on which the weighted scoring model was built. These methods are described in detail elsewhere [14] but briefly, the opinions of the three experts were combined using probabilistic techniques which took into account not only differences between responders but also differences in diagnostic judgement thresholds between the experts. The result of applying these techniques was a number between 0 and 1 for each questionnaire in the reviewed phase 1 subset which reflected probability of asthma of the respondent. Those with probability greater than 0.5 were designated 'asthmatic'.

Since the aim of a screening system is generally to identify individuals with a high probability of the condition being tested for, the performance of the screening system on high probability individuals is most important. For this reason it was necessary in this study to identify a set of high probability asthmatics for clinical review in the phase 3 validation stage. It was also necessary that these high probability asthmatics were identified by a system which was independent of the weighted scoring system being tested. For this reason, a neural network was used to rank the 2001 survey questionnaires in order of probability of asthma. The neural network is a sophisticated statistical technique that was used to model complex relationships between questions on the questionnaire to predict probability of asthma of a respondent based on questionnaire responses. The neural network model was validated [20] and applied to the questionnaires from the 2001 population survey to produce a population ranking based on individual predicted probability of asthma according to the neural network model. The top 10% most likely asthmatics from this population ranking, along with some low probability individuals, comprised the independent subset used to test the weighted scoring system in phase 3.

The question weights were consistent with clinical explanations. Wheeze in the last twelve months was the most important symptom and the weights for each age band decreased with increasing age which is consistent with the explanation that wheezing in older people can be explained by reasons other than asthma, for example, heart disease, malignant lung disease or one of the chronic obstructive lung conditions collectively referred to as COPD. The interpretation of the low weighting given to respiratory symptoms other than wheeze, for example, night cough or shortness of breath, is that these symptoms are highly correlated with wheezing, and as such provide no additional information over an above the high 'wheeze' weighting. Whereas any of these symptoms viewed in isolation would be highly correlated with a diagnosis of asthma, when viewed in the context of a multivariate model such as this weighted scoring system, it is the interaction between the variables which defines the final model. The small negative coefficient for smoking is consistent with the explanation that those with asthma tend to stop smoking or never to begin. Alternatively, asthma-like symptoms in some smokers may be caused by conditions other than asthma, for example malignant lung disease, COPD, or heart disease, or smoking itself may cause symptoms.

The intended application of weighted scoring is to find individuals who are likely to have asthma but are not already receiving appropriate treatment. Superficially, it may appear that the questions relating to an attack of asthma in the last twelve months and current medication for asthma should be excluded from the model as they may produce higher scores in asthmatics who are already diagnosed and receiving treatment, effectively disadvantaging the undiagnosed individuals. However, the relationship between these two questions and practice records of diagnosis is far from definitive. For example in the 1995 survey, of the 6570 respondents who had no record of either diagnosis of asthma nor medication prescription in the last year, 175 individuals answered 'Yes' to the current medication question on the questionnaire and 128 reported an asthma attack in the last twelve months. The scoring system reflects an accurate ranking of the whole population and the questions relating to attack of asthma in the last 12 months and current medication for asthma were found to be important in the whole population model. In general, higher scores reflected higher PPV estimates, but the final column of table 2 illustrates that this is not a simple linear relationship, rather the scores can be considered associated into four priority levels, that is, 9 or more, 7–8, 5–6, and 4 or lower.

One possible source of error in the weighted scoring system is under or over self-reporting of symptoms, such as the individual ranked as low probability who was found clinically asthmatic in the absence of reported symptoms on the questionnaire. Another possible source of error is differences between the development data sample and the intended target population due to non-response in the postal survey, exclusion of incomplete questionnaires and non-attendance at clinical review. Analyses revealed no evidence of sample bias from these sources in terms of gender or numbers of 'yes' answers to the six key questions on the questionnaire. Those who attended for review had a higher median age (50 years) than those invited but not attending (32 years). It is also important to acknowledge that the system here was tested using postal questionnaires, whereas in practice the patient may well complete the questionnaire in a surgery or clinic environment.

The question weights were derived from a logistic regression model and commonly, scarcity of data means that this modelling technique is applied as a single model fitted to the entire data subset [21] with none reserved for independent validation and no allowance made for differences in prevalence between training data and the general population. However, this gives limited information about how well the model will generalise to the wider community. In this study three steps were taken to gain a realistic estimate of model performance. First, the regression coefficients were not based on a single model but were the average of several representative models [22]. Second, there was an independent validation set comprising examples from the higher scores likely to be of interest when targeting diagnostic examinations, along with a small number of respondents believed to have a low probability of asthma. Third, the consensus probability of asthma information available for the phase 1 reviewed individuals allowed the positive predictive values estimated in phase 2 to take account of differences in prevalence of asthma between the questionnaires used in developing the scoring system and the general population. However, the intended target population is practice attendees rather than a population-wide survey and prevalence of all diseases including asthma may be higher in practice attendees than in the general population. In this case the positive predictive values reported in phase 2 may be under-estimates.

Assessing likelihood of asthma and ranking by simply counting the number of 'yes' answers to key questions is a simple but relatively crude method since it assumes equal weights for the questions. However, some responses may exert a stronger influence than others, there may be interactions between responses and features such as age and gender cannot be included at all. For example, an elderly person with four 'yes' answers to the key questions may be considered less likely to be asthmatic than a young person with three, since respiratory symptoms in the elderly may sometimes be explained by conditions other than asthma. Hence, ranking according to the number of 'yes' answers may not produce the required probability ordering. This is illustrated by the small differences in proportions of clinical asthmatics associated with the various simple score thresholds shown in table 4.

Where the availability of resources is a limiting factor, the relevant measure of effectiveness in targeting clinical review is the 'true positive' rate, that is, the percentage of individuals scoring above the threshold in whom the diagnosis is confirmed. The other commonly reported test measures of sensitivity, specificity and negative predictive value have no equivalent in a technique based on population ranking where the aim is to target expensive clinical examinations to those most at risk and minimise the 'wasted' examination of false positives.

## Conclusions

When resources for diagnostic examination are limited prioritisation may be necessary. The use of weighted scoring and priority levels rather than the more conventional binary separation into positive and negative allows the score threshold to be adjusted to balance patient need with available resources

## Abbreviations

COPD Chronic obstructive pulmonary disease

OAD Obstructive airways disease

PPV Positive predictive value

WYCAP Wythenshawe Community Asthma Project

## Competing interests

The author(s) declare that they have no competing interests.

## Authors' contributions

SH performed the statistical analysis and developed the weights. TF and PF performed the clinical reviews. JS advised on statistical aspects of combining expert opinions. MH managed the data collection. All authors participated in study design and read and approved the final manuscript.

**Table 5:** Information for estimating PPV for those scoring 9 or more in the 2001 population survey

Stratum (*i*) (number of 'yes' answers to key questions)	Score (*j*)	Number of respondents	ω_*i*j _proportion of population postal questionnaires in stratum *i*,	∏'_*ij *_prevalence of asthma for score j in stratum i	ω_*i*j _× ∏'_*ij*_
6	12	2	0.0250	0.9914	0.0248
6	11	6	0.0750	0.9914	0.0744
6	10	5	0.0625	0.9867	0.0616
6	9	7	0.0875	0.7996	0.0700
5	12	1	0.0125	0.7181	0.0090
5	11	3	0.0375	0.7181	0.0269
5	10	9	0.1125	0.9777	0.1100
5	9	16	0.2000	0.8938	0.1788
4	10	3	0.0375	0.8908	0.0334
4	9	11	0.1375	0.9975	0.1371
1 – 3	11	1	0.0125	0.9633	0.0120
1 – 3	10	3	0.0375	0.9757	0.0366
1 – 3	9	13	0.1625	0.9880	0.1606
Total		80	1		

Estimated PPV for those scoring 9 or more:

## Pre-publication history

The pre-publication history for this paper can be accessed here:



## Supplementary Material

Additional File 1 The Respiratory Questionnaire. The respiratory questionnaire used in the postal survey Click here for file

Additional File 2 Estimating PPV for weighted scores. Details of method used for estimating PPV based on postal questionnaire responses Click here for file

## References

[B1] Weiss KB (1996). An overview of recent trends in asthma epidemiology. Eur Respir Rev.

[B2] Omran M, Russell G (1996). Continuing increase in respiratory symptoms and atopy in Aberdeen schoolchildren. BMJ.

[B3] Kaur B, Anderson HR, Austin J (1998). Prevalence of asthma symptoms, diagnosis and treatment in 12 to 14 year old children across Great Britain (International study of asthma and allergies in childhood (ISAAC UK). BMJ.

[B4] De Marco R, Cerven I, Bugiani M, Ferrari M, Berlato G (1998). An undetected burden of asthma in Italy: the relationship between clinical and epidemiological diagnosis of asthma. Eur Resp J.

[B5] Speight ANP, Lee DA, Hey EN (1983). Underdiagnosis and undertreatment of asthma in childhood. BMJ.

[B6] Pederson S (1997). Early use of inhaled steroids in children with asthma. Clin Exp Allergy.

[B7] (2002). Global Strategy for asthma management and prevention. Bethesda, National Heart, Lung and Blood Institute NIH Publication Number 02-3659.

[B8] Frank PI, Ferry S, Moorhead T, Hannaford P (1996). Use of a postal questionnaire to estimate the likely under-diagnosis of asthma-like illness in adults. Br J Gen Pract.

[B9] Frank TL, Frank PI, McNamee, Wright T, Hannaford P, Morrison J (1999). Assessment of a simple scoring system applied to a screening questionnaire for asthma in children aged 5–15 years. Eur Respir J.

[B10] Frank TL, Frank PI, Cropper JA, Hazell M, Hannaford P, McNamee R, Hirsch S, Pickering CAC (2003). Identification of adults with symptoms suggestive of obstructive airways disease: Validation of a postal respiratory questionnaire. BMC Family Practice.

[B11] Burney PGI, Luczynska C, Chinn, Jarvis D (1994). The European community respiratory health survey. Eur Resp J.

[B12] SPSS (2001). Version 1010 for Windows Chicago: SPSS Inc.

[B13] Tarassenko LA (1998). A Guide to Neural Computing Applications.

[B14] Hirsch S, Shapiro JL, Turega MA, Frank TL, Niven RM, Frank PI (2001). Using a Neural Network to Screen a Population for Asthma. Annals of Epidemiology.

[B15] Hirsch S, Frank PI, Shapiro J (1997). Use of an artificial neural network in estimating prevalence and assessing under-diagnosis of asthma. Neural Computing & Applications.

[B16] Pickles A, Dunn G (1995). Screening for stratification in two-phase ('two stage') epidemiological surveys. Stat Methods Med Res.

[B17] Bishop CM (1995). Neural Networks for Pattern Recognition.

[B18] Hirsch S, Shapiro J, Frank P, Kluwer Academic Publishers (2003). The application of neural network systems techniques to asthma screening and prevalence estimation. In Computational Methods in Biophysics, Biomaterials, Biotechnology and Medical Systems, Diagnostic Methods.

[B19] Redline S, Gruchalla RS, Wolf RL, Yawn BP, Cartar L, Gan V, Nelson P, Wollan P (2004). Development and validation of school-based asthma and allergy screening questionnaires in a 4-city study. Ann Allergy Asthma Immunol.

[B20] Hirsch S, Frank TL, Hazell M, Frank PI (2005). Screening for Asthma by Population Ranking: A Validation Study. Annals of Epidemiology.

[B21] Thiadens HA (1998). Identifying asthma and chronic obstructive pulmonary disease in patients with persistent cough presenting to general practitioners: descriptive study. BMJ.

[B22] Tarassenko LA (1998). A Guide to Neural Computing Applications.

