# Supplementary material for: Development of a questionnaire weighted scoring system to target diagnostic examinations for asthma in adults: a modelling study
Source: BMC Fam Pract. 2004 Dec 17;5:30. doi: 10.1186/1471-2296-5-30 (PMC545076; doi:10.1186/1471-2296-5-30)
Supplement: Additional File 1 — The Respiratory Questionnaire. The respiratory questionnaire used in the postal survey [file 1471-2296-5-30-S1.doc]

APPENDIX 1

**The respiratory questionnaire**

**(The key questions were 3,4,5,6,9,10)**

Please tick the appropriate box

1. What is your date of birth?

2. Are you FEMALE MALE

3. Have you had wheezing or whistling in your chest NO YES

at any time in the last 12 months?

IF ‘NO’ GO TO QUESTION 4,

IF ‘YES’:

3.1 Have you been at all breathless when the wheezing NO YES

noise was present?

3.2 Have you had this wheezing or whistling when NO YES

you did not have a cold?

4. Have you woken up with a feeling of tightness in NO YES

your chest in the last 12 months?

5. Have you been woken by an attack of shortness of NO YES

breath at any time in the last 12 months?

6. Have you been woken by an attack of coughing at NO YES

any time in the last 12 months?

7. Have you had an attack of asthma in the last 12 months? NO YES

8. Are you currently taking any medicine for asthma? NO YES

(including inhalers, aerosols or tablets)

9. Has any person in your family (parents, grandparents, NO YES

sisters, brothers, or your children) had asthma?

10. Have you ever had hayfever or eczema? NO YES

11. How many cigarettes do you smoke each day? ..........per day

12. How many other adults live in your house?

How many of these adults smoke?

THANK YOU FOR YOUR HELP

PLEASE RETURN THIS FORM TO US IN THE REPLY-PAID ENVELOPE
