# Supplementary material for: Development of a questionnaire weighted scoring system to target diagnostic examinations for asthma in adults: a modelling study
Source: BMC Fam Pract. 2004 Dec 17;5:30. doi: 10.1186/1471-2296-5-30 (PMC545076; doi:10.1186/1471-2296-5-30)
Supplement: Additional File 2 — Estimating PPV for weighted scores. Details of method used for estimating PPV based on postal questionnaire responses [file 1471-2296-5-30-S2.doc]

APPENDIX 2

## Estimating PPV for weighted scores

PPV is the probability that the subject has the disorder given that he/she screens positive (weighted score higher than chosen threshold score).

Using standard 2-phase survey techniques [15], in a stratified population, the population asthma prevalence ($P$) is given by

*(1)*

where s is the number of population strata,

$w\text{ }i$ is the proportion of population postal questionnaires in stratum *i,*

and is the population prevalence of asthma in stratum *i*.

Considering each population stratum separately, the scores give the more refined prevalence estimate

*(2)*

where s is the number of strata,

*r*  is the number of scores appearing in stratum *i,*

$w\text{ }i\text{ j}$ is the proportion of postal questionnaires with score *j* in stratum *i,*

and is the prevalence of asthma in respondents with score *j* in stratum *i*

is estimated as the prevalence $\Pi'ij$ of asthma in the corresponding score in the corresponding stratum of the 1995 stratified random sample, inferred from the known probability of asthma of the subjects.

Hence PPV for any screening threshold was estimated as

*(3)*

for all respondents with the threshold score or higher.

Example: Estimating PPV for undiagnosed respondents scoring 9 or more in the 2001 survey

# Number of respondents scoring 9 or more = 80

As shown in table 5, 2 respondents had 6 ‘yes’ answers to the key questions and a weighted score of 12, 6 respondents had 6 ‘yes’ answers to the key questions and a weighted score of 11, and so on.

Hence, estimated PPV for score of 9 or more is

##### = 0.9352
